# Injury Analysis and Prevention in Vehicle Safety

**DOI:** 10.1155/2018/9532598

**Published:** 2018-10-04

**Authors:** Jun Xu, Jikuang Yang, Jingwen Hu, Matthew Panzer, Yong Peng

**Affiliations:** ^1^Beihang University, Beijing, China; ^2^Chalmers University of Technology, Göteborg, Sweden; ^3^University of Michigan, Ann Arbor, MI, USA; ^4^University of Virginia, Charlottesville, VA, USA; ^5^Central South University, Changsha, China

Injury and injury prevention of the drivers, passengers, and vulnerable road users are one of the most important issues in vehicle accidents and cannot be overlooked. The challenges of this issue focus on the following aspects: (a) the accident conditions causing injuries and mechanisms of these injuries; (b) the effect of characteristics of an injured person, e.g., age, gender, and types of vulnerable road users, and the development of computational models for the injured-included accidents; and (c) the methods and designs for injury prevention. In this special issue on injury analysis and prevention in vehicle safety, we have invited a few papers that address such issues.

The first paper (S. Guan et al.) develops experiments to study the thoracoabdominal injuries suffered from caudocephalad impacts using pigs. 21 adult minipigs divided into three groups are tested in different impact velocities, and the mechanical responses are then analyzed. The second paper (Y. Yan et al.) investigates the effect of neck muscle active force on whiplash injury of the cervical spine to indicate that the neck active force influenced the head-neck dynamic response and whiplash injury during a collision, especially in a low-speed collision. The third paper (F. Li et al.) studies the effect of the neck muscle activation on head and neck injuries to find out that the activation of neck muscles can lower not only the head horizontal acceleration under different impact intensities but also the head angular acceleration in medium- and high-speed impacts. The forth paper (J. Zheng et al.) uses a numerical method to study the risk factors affecting lumbar spine injuries. The effects of coefficient of friction, impact velocity, cushion thickness and stiffness, and cushion angle on the risk of lumbar spine injuries are analyzed.

The fifth paper (Y. Peng et al.) investigates the dynamic response and head injuries of standing subway passengers during collisions based on the MADYMO models to provide guidance for the safety design of the subway and some advices for standing subway passengers. The sixth paper (T. Zou et al.) analyzes the injury of the riders upon car-electric bicycle accident. After accident reconstruction, data acquisition, data verification, and screening of 57 car-electric bicycle accidents wherein riders are hit to the engine hood and thrown to the air, the data obtained from the remaining 53 cases were analyzed. The seventh paper (S. Shang et al.) focuses on the head and brain injuries of one new vulnerable road user which is a rider using electric self-balancing scooters. The eighth paper (J. Huang et al.) develops the age-specific lower extremity finite element model for simulating pedestrian accidents. The development of the age-specific lower extremity models will lead to an improved understanding of the pedestrian lower extremity injury mechanisms and injury risk prediction for the whole population in vehicle-pedestrian collision accidents.

The ninth paper (H. Li et al.) analyzes the effects of curtain airbag (CAB) on occupant kinematics and injury indexes in a rollover crash. The simulation results indicated that the occupant kinematics and most injury indexes were improved with the help of CAB in such rollover scenario. The tenth paper (G. Zhang et al.) investigates the relationships between the front-end styling features of SUVs and head injuries at the styling design stage for pedestrian protection performance improvement and product development efficiency. The eleventh paper (F. Mo et al.) designs a conceptual bumper energy absorber coupling pedestrian safety and low-speed impact requirements. The new X-shape energy absorber can meet both pedestrian safety and low-speed impact requirements well by altering the main deformation modes according to different impact energy levels.

